# Long Non-Coding RNA 1810026B05Rik Mediates Cerebral Ischemia/Reperfusion-Induced Neuronal Injury Through NF-κB Pathway Activation

**DOI:** 10.3390/ijms26199756

**Published:** 2025-10-07

**Authors:** Hao Zhang, Meng Li, Jiayu Yao, Xuan Jiang, Junxiao Feng, Xingjuan Shi, Xiaoou Sun

**Affiliations:** 1 Institute of Biomedical and Pharmaceutical Sciences, Guangdong University of Technology, Guangzhou 510006, China; lm135837@163.com; 2 Key Laboratory of Developmental Genes and Human Disease, School of Life Science and Technology, Southeast University, Nanjing 210096, China; jiayuyaoyjy@163.com (J.Y.); junxiaofeng08@163.com (J.F.); xingjuanshi@seu.edu.cn (X.S.)

**Keywords:** cerebral ischemia, lncRNA-1810026B05Rik, IκBα phosphorylation, NF-κB

## Abstract

Cerebral ischemia/reperfusion (I/R) injury remains a significant contributor to adult neurological morbidity, primarily due to exacerbated neuroinflammation and cell apoptosis. These processes amplify brain damage through the release of various pro-inflammatory cytokines and pro-apoptotic mediators. Although long non-coding RNAs (lncRNAs) are increasingly recognized for their involvement in regulating diverse biological pathways, their precise role in cerebral I/R injury has not been fully elucidated. In the current study, transcriptomic profiling was conducted using a rat model of focal cerebral I/R, leading to the identification of lncRNA-1810026B05Rik—also referred to as CHASERR—as a novel lncRNA responsive to ischemic conditions. The elevated expression of this lncRNA was observed in mouse brain tissues subjected to middle cerebral artery occlusion followed by reperfusion (MCAO/R), as well as in primary cortical neurons derived from rats exposed to oxygen-glucose deprivation and subsequent reoxygenation (OGD/R). The results suggested that lncRNA-1810026B05RiK mediates the activation of the nuclear factor-kappaB (NF-κB) signaling pathway by physically binding to NF-kappa-B inhibitor alpha (IκBα) and promoting its phosphorylation, thus leading to neuroinflammation and neuronal apoptosis during cerebral ischemia/reperfusion. In addition, lncRNA-1810026B05Rik knockdown acts as an NF-κB inhibitor in the OGD/R and MCAO/R pathological processes, suggesting that lncRNA-1810026B05Rik downregulation exerts a protective effect on cerebral I/R injury. In summary, the lncRNA-1810026B05Rik has been identified as a critical regulator of neuronal apoptosis and inflammation through the activation of the NF-κB signaling cascade. This discovery uncovers a previously unrecognized role of 1810026B05Rik in the molecular mechanisms underlying ischemic stroke, offering valuable insights into disease pathology. Moreover, its involvement highlights its potential as a novel therapeutic target, paving the way for innovative treatment strategies for stroke patients.

## 1. Introduction

Over the past years, there has been a rise in stroke prevalence due to lifestyle alterations and the elderly demographic shift. Stroke has emerged as the primary reason for disability and ranks as the second most lethal ailment in China [1]. Ischemic stroke, which accounts for roughly 70% of all stroke cases, is the most common type of stroke [2]. Ischemic stroke refers to the necrosis of brain tissue caused by insufficient blood supply to the cerebrum resulting from the narrowing or complete blockage of the artery supplying blood to the brain. Currently, there are limited effective therapies available for treating cerebral ischemia, with the primary approach being thrombolytic therapy. Thrombolytic therapy, which primarily involves the use of tissue plasminogen activator (tPA), is the only approved treatment for ischemic stroke [3]. However, while tPA restores blood flow, the rapid reperfusion can also worsen the injury, leading to additional damage in the affected brain tissue. Cerebral ischemia/reperfusion injury (CIRI) can lead to various outcomes, including but not limited to free radical harm, swelling of cells, apoptosis, and necrosis [4]. Cerebral ischemia/reperfusion (I/R) can lead to neuronal apoptosis and necrosis, which is a major change in CIRI [5]. Research has provided evidence demonstrating that the primary reason for secondary brain damage subsequent to cerebral ischemia is the exaggerated inflammatory reaction within the ischemic penumbra and inhibiting the overexpression of inflammatory mediators can alleviate secondary brain injury after cerebral ischemia [6]. However, effective CRI treatment remains a challenge.

The research has provided evidence that the significant involvement of regulatory regions in diverse biological processes within the non-coding RNA (ncRNA) segment of the genome [7]. To our current knowledge, only a small fraction, approximately 1.2–1.5%, of the genome is responsible for encoding proteins, while the majority of non-coding regions serve as regulatory elements that are transcribed into ncRNA [8,9]. Specifically, a type of ncRNA known as lncRNA, which surpasses the length of 200 nucleotides, induces alterations in gene transcription through the mechanism of epigenetic regulation [10,11,12]. Previous studies have found increased autophagy and lncRNA-MALAT1 of hippocampal neurons in epileptic patients [13]. Several lncRNAs exhibited distinct expression patterns in blood specimen obtained from patients with acute ischemic stroke, cortical tissues of mice, and neurons following ischemia/reperfusion (I/R) [14,15]. Hence, the application prospects of lncRNA as a potential biomarker for diagnosing and treating ischemic stroke are significant. Recent findings demonstrate the regulation of 147 lncRNAs, with a focus on lncRNA-1810026B05Rik, in primary brain microvascular endothelial cell (BMECs) isolated from brain tissue, upon exposure to oxygen-glucose deprivation (OGD) for 16 h [16]. Moreover, a shared signaling pathway between BMECs and early developmental neurons suggests neuronal lncRNA-1810026B05Rik’s possible involvement in regulating CIRI [17]. Nonetheless, the mechanism by which I/R damage is caused by ischemic stroke has remained unclear.

Research studies have identified that lncRNA exerts an influence on the transcription of promoters associated with coding genes, subsequently disrupting the expression of genes located downstream [18,19]. Furthermore, lncRNA possesses the ability to disrupt the mRNA cleavage process while also instigating various types of cleavage [20]. Earlier research has revealed a close association between neuronal inflammation, apoptosis, and brain tissue impairment subsequent to a stroke [21,22]. Neurons that have suffered damage release a substantial quantity of cytokines, including TNF-α, IL-1β, Bax, and caspase-3, consequently fostering the infiltration of neutrophils, and macrophages into the cortex affected by the infarct [23]. In addition, the immune response and apoptosis response upon infection are regulated by NF-κB, which is crucial [24]. Activation of the NF-κB pathway occurs during CIRI. Past research has indicated that neuron survival is promoted and the apoptosis of neurons is inhibited by inhibiting the NF-κB pathway, thereby providing protection against CIRI [25]. In experimental models of ischemic animals, numerous researchers have noted heightened levels of pro-inflammatory factors leading to apoptosis. Additionally, they have observed enhanced transcription activity of nuclear NF-κB [26]. The inhibition of the NF-κB pathway in mice with a p50 gene knockout results in a reduction in the size of the infarction and facilitates the enhancement of functional recovery [27]. These reports indicate that inhibition of inflammatory apoptotic response and NF-κB pathways could be potential therapeutic targets for CIRI.

This study confirmed the production of lncRNA-1810026B05Rik during cerebral ischemia/reperfusion injury. Our research demonstrates that lncRNA-1810026B05Rik has a significant impact on CIRI by modulating the NF-κB pathway. Specifically, lncRNA-1810026B05Rik promotes NF-κB pathway activation and regulates the phosphorylation of IκBa through direct binding. This interaction subsequently leads to enhanced expression and secretion of inflammatory cytokines such as TNF-α, IL-1β, and IL-6, as well as pro-apoptotic factors like Caspase-3, caspase-9, and Bax. Consequently, in both in vivo and in vitro models, these alterations induce detrimental effects such as apoptosis and cell death, contributing to I/R damage. Therefore, targeting lncRNA-1810026B05Rik could hold therapeutic potential for stroke treatment.

## 2. Results

### 2.1. lncRNA-1810026B05Rik Was Significantly Increased After Ischemic/Reperfusion (I/R) In Vivo and In Vitro

**Figure 1:**
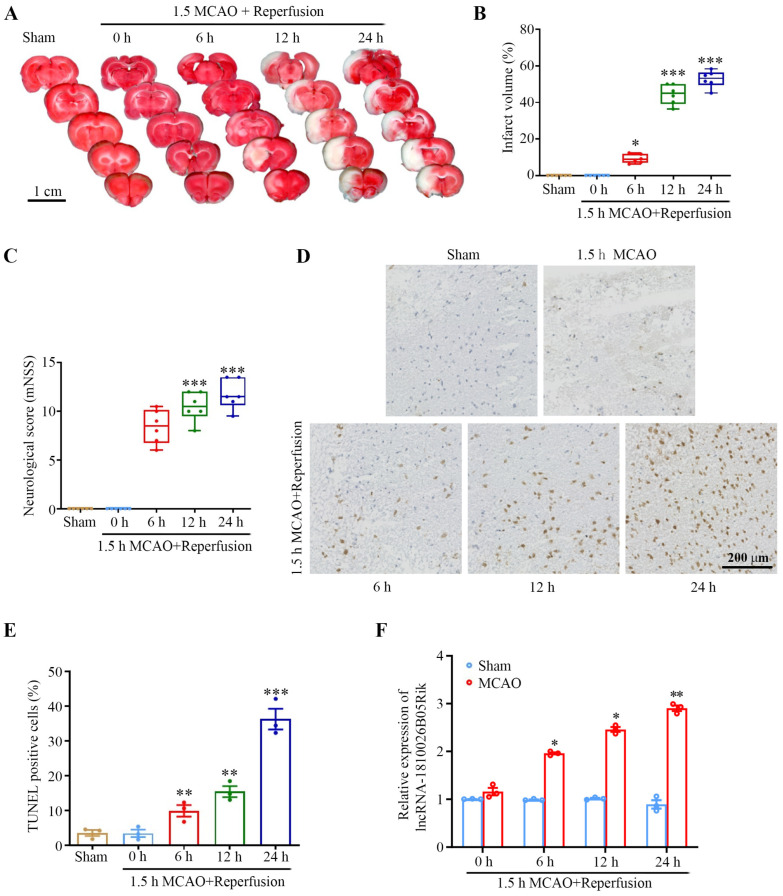
In vivo involvement of lncRNA-1810026B05Rik in I/R-induced brain injury. (**A**) Representative TTC-stained brain sections showing infarct areas (magenta: viable tissue; white: infarcted regions). Scale bar = 1 cm. (**B**) Infarct volume in the brain was quantitatively measured following I/R injury (*n* = 6). (**C**) Neurological deficits were evaluated using the modified Neurological Severity Score (mNSS) after I/R (*n* = 6). (**D**,**E**) Neuronal apoptosis was analyzed I/R using TUNEL staining. Scale bar: 200 μm (*n* = 3). (**F**) Temporal expression profile of lncRNA-1810026B05Rik in mouse brain tissue at 0, 6, 12, and 24 h after MCAO, as determined by qRT-PCR (*n* = 3). All data are represented as mean ± SD. One way ANOVA, * *p* < 0.05, ** *p* < 0.01, *** *p* < 0.001 versus Sham group. I/R, ischemia/reperfusion; TTC, triphenyl tetrazolium chloride; mNSS, Neurological Severity Score; qRT-PCR, quantitative real-time polymerase chain reaction; MCAO, cerebral artery occlusion.

**Figure 2:**
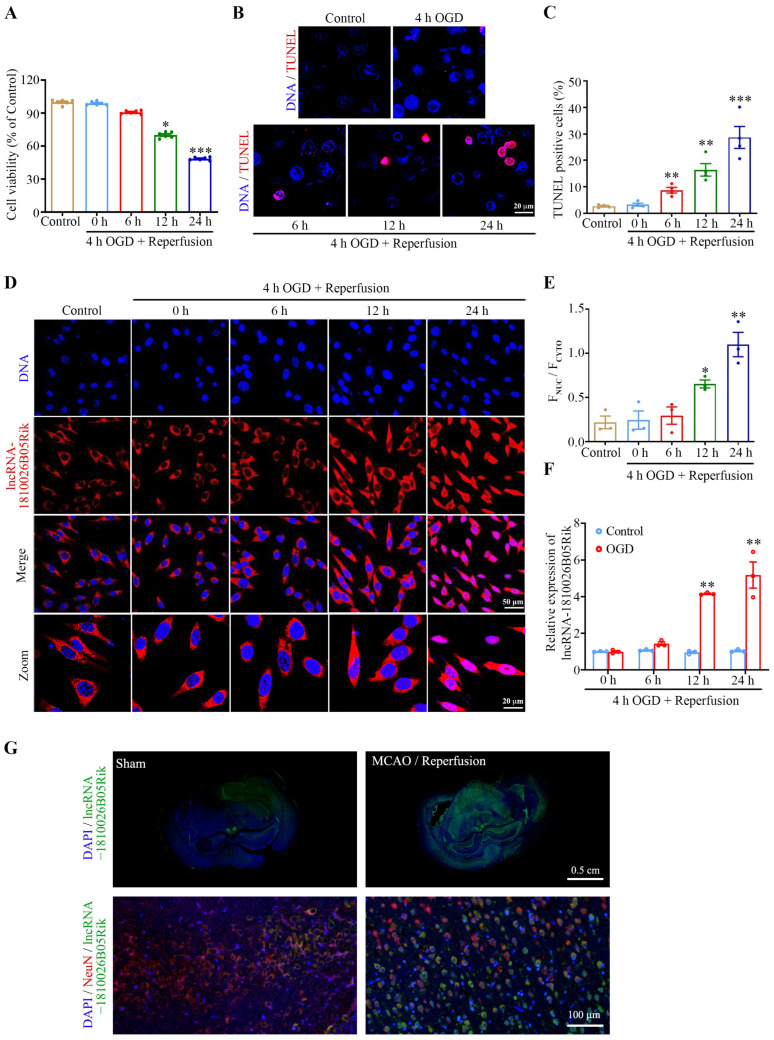
Subcellular distribution and expression analysis of lncRNA-1810026B05Rik under in vitro I/R-induced hypoxic stress. (**A**) Neuronal viability following OGD/R was assessed using the CCK-8 assay (*n* = 6). (**B**,**C**) Apoptotic cells were visualized using TUNEL staining post-I/R injury, with nuclei counterstained by Hoechst 33342 (blue) (*n* = 4). Scale bar: 20 μm. (**D**) RNA FISH images illustrating the subcellular localization of lncRNA-1810026B05Rik in neurons. Nuclei were counterstained with Hoechst 33342 (blue) (*n* = 3). Scale bars: 50 μm and 20 μm. (**E**) Subcellular localization fluorescence intensity of 1810026B05Rik in different lesions induced by OGD/R. (**F**) 1810026B05Rik expression was detected by qRT-PCR at each time point in cultured neurons subjected to 1 h of OGD and 0, 6, 12, or 24 h of reoxygenation (*n* = 3). (**G**) Representative RNA FISH images showing the intracellular localization of lncRNA-1810026B05Rik in rat brain, Nuclei (blue) were counterstained with Hoechst 33342 (*n* = 3). Scale bar: 0.5 cm/100 μm. All data are represented as mean ± SD. One way ANOVA, * *p* < 0.05, ** *p* < 0.01, *** *p* < 0.001 versus Control group. OGD, oxygen-glucose deprivation; OGD/R, oxygen-glucose deprivation/reoxygenation; RNA FISH, RNA fluorescence in situ hybridization.

To investigate the potential significance of lncRNA-1810026B05Rik in I/R injury, we established both an in vivo model of middle cerebral artery occlusion/reperfusion (MCAO/R) in rats and an in vitro model of oxygen-glucose deprivation/reperfusion (OGD/R) in neurons. Subsequently, we conducted TTC staining, mNSS assessment, CCK8 assay, and TUNEL assay to evaluate the extent of brain infarction, neurological function, cell viability, and cell apoptosis, respectively. The results showed that after 6 h of reperfusion, the damage to the rats’ brains increased significantly with the increase in reperfusion time (Figure 1A–E). We conducted detection of the expression of lncRNA-1810026B05Rik in samples of ischemic brain tissue and neurons at 0, 6, 12, 24, and 48 h after establishing I/R. The findings revealed a significant increase in the level of lncRNA-1810026B05Rik at 6 h post MCAO/R model (Figure 1F). In addition, after 6 h of reoxygenation, the damage to the cultured primary rat neurons increased significantly with the increase in reoxygenation time (Figure 2A–C), and the qRT-PCR results revealed that the expression of lncRNA-1810026B05Rik began to increase 6 h after OGD/R (Figure 2F). These findings indicate that lncRNA-1810026B05Rik may play an improtant role in I/R injury.

### 2.2. The Subcellular Localization of lncRNA-1810026B05Rik on OGD/R-Induced Hypoxic Injury in Primary Rat Neurons

The cellular positioning of lncRNA commonly contributes significantly to its biological operation. Hence, the RNA FISH approach was employed to examine the neuron positioning of lncRNA-1810026B05Rik at various time intervals after hypoxia-reoxygenation. As depicted in Figure 2D,E, in typical conditions, the lncRNA-1810026B05Rik transcripts are dispersed within the cytoplasm of neurons. Following OGD/R prolongation, the lncRNA-1810026B05Rik transcript commenced accumulating in the neuronal nucleus (Figure 2F). In addition, morphological data on the expression of lncRNA-1810026B05Rik in brain tissue were further examined. As shown in Figure 2G. LncRNA-1810026B05Rik is expressed in various cell types of brain tissue. Compared to sham group, the fluorescence intensity of lncRNA-1810026B05Rik was remarkably increased in the I/R group. We found that lncRNA-1810026B05Rik co-localized with NeuN, and in the MCAO/R group, more cytoplasmic lncRNA-1810026B05Rik translocated to the cell nucleus. We also observed an increased intensity of lncRNA-1810026B05Rik in the cell nucleus of the MCAO/R group, particularly at high magnification. These results are consistent with the cytological data. Therefore, the cytoplasm of undamaged tissues primarily expresses lncRNA-1810026B05Rik, and upon I/R injury, it relocates to the nucleus. The findings imply that lncRNA-1810026B05Rik participates in the pathological process following I/R and undergoes nuclear translocation.

### 2.3. Effect of lncRNA-1810026B05Rik on OGD/R-Induced Hypoxic Injury in Neurons

**Figure 3:**
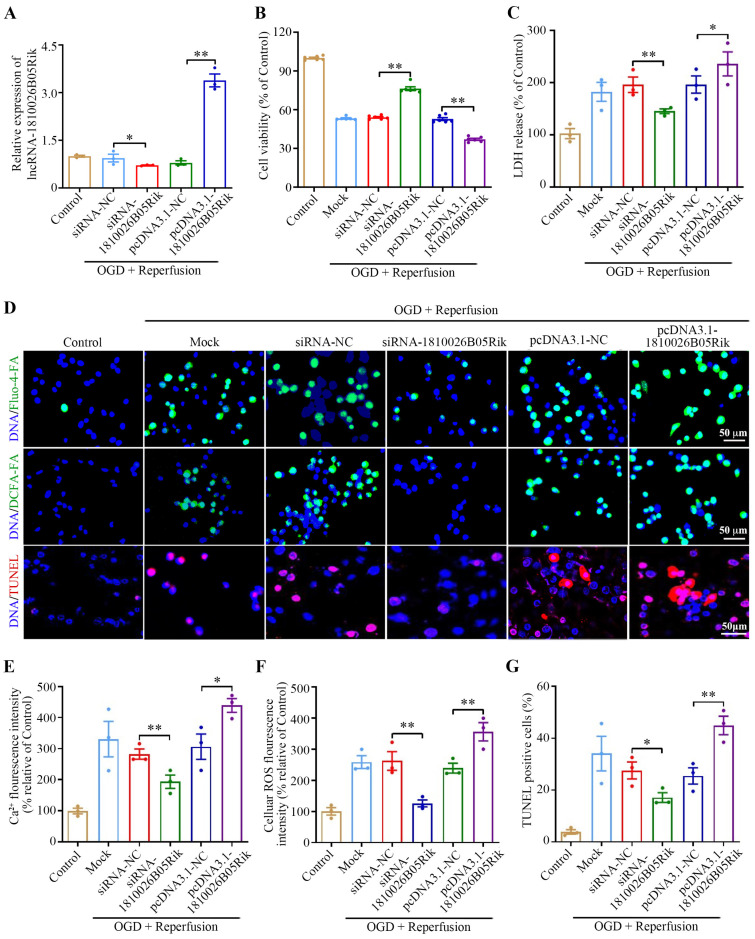
Impact of lncRNA-1810026B05Rik on OGD/R-induced neuronal injury. Primary neurons were transfected with siRNA-NC, siRNA targeting 1810026B05Rik, pcDNA3.1-NC, or pcDNA3.1-1810026B05Rik, followed by OGD/R for 4 h and reoxygenation for 24 h. (**A**) qRT-PCR analysis confirmed the altered expression of 1810026B05Rik post-transfection (*n* = 3). (**B**) Cell viability was assessed using the CCK-8 assay (*n* = 3). (**C**) LDH release was measured as an indicator of neuronal injury (*n* = 3). (**D**) After 24 h of reoxygenation, the intracellular Ca^2+^ concentration, ROS levels and apoptosis of neurons were determined using Fluo-4 AM, DCFH-DA or TUNEL staining, Nuclei (blue) were counterstained with Hoechst 33342, respectively. Scale bar: 50 μm. (**E**) The intracellular Ca^2+^ fluorescence intensity, demonstrated as the green fluorescence ratio, was calculated (*n* = 3). (**F**) The ROS fluorescence intensity was analyzed (*n* = 3). (**G**) The apoptotic neurons shown as TUNEL-positive cells were calculated (*n* = 3). All data are represented as mean ± SD. One way ANOVA, * *p* < 0.05, ** *p* < 0.01 versus siRNA-NC or pcDNA3.1-NC group. NC: negative control; LDH, lactate dehydrogenase; DCFH-DA, dichloro-dihydro-fluorescein diacetate; ROS, reactive oxygen species.

To illustrate the effect of lncRNA-1810026B05Rik, neurons with short interfering RNA or pcDNA3.1-1810026B05Rik plasmid were transfected to interfere with or increase the levels of lncRNA-1810026B05Rik. Neuronal assessments were conducted to evaluate cell viability, LDH release and ROS production following OGD/R. As illustrated in Figure 3A, the expression of lncRNA-1810026B05Rik was altered-either upregulated or downregulated-in both untreated and OGD/R-exposed neurons relative to their respective controls. Silencing lncRNA-1810026B05Rik led to a marked reduction in neuronal viability (Figure 3B) and significantly elevated LDH release following OGD/R treatment (Figure 3C), indicating increased cell injury. As ROS are central to mitochondrial dysfunction, and intracellular calcium accumulation is a well-known trigger of apoptotic cascades, we further explored these parameters. Knockdown of lncRNA-1810026B05Rik resulted in a significant decrease in intracellular Ca^2+^ levels (Figure 3D,E), and remarkably, it led to a significant reduction in ROS production (Figure 3D,F). In addition, neuroprotection was observed in terms of reduced neuronal apoptosis, as detected by TUNEL staining (Figure 3D,G). In contrast, overexpression of this lncRNA reversed these effects, mitigating oxidative stress and apoptotic injury. Collectively, these findings suggest that suppression of lncRNA-1810026B05Rik exerts a neuroprotective effect by reducing ROS levels, calcium influx, and apoptosis, ultimately attenuating OGD/R-induced cellular damage and positioning lncRNA-1810026B05Rik as a potential modulator in ischemic injury.

### 2.4. lncRNA-1810026B05Rik Contributed to NF-κB Activation in OGD/R-Treated Neurons

**Figure 4:**
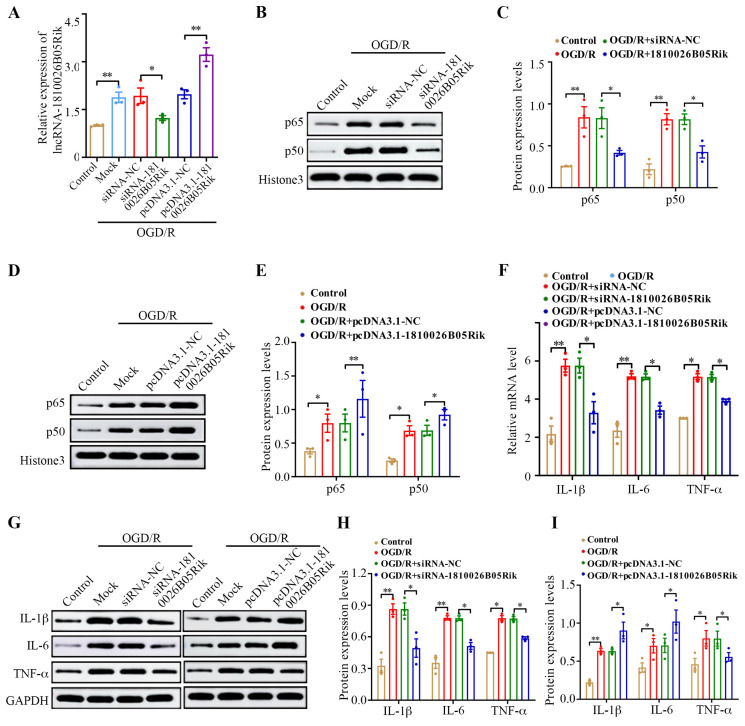
lncRNA-1810026B05Rik promotes NF-κB pathway activation in neurons subjected to OGD/R. (**A**) The expression of 1810026B05Rik was evaluated by qRT-PCR in untreated neurons and in OGD/R-treated cells transfected with specific siRNAs or expression plasmids (*n* = 3). (**B**,**D**) Nuclear levels of NF-κB subunits p65 and p50 were analyzed by Western blot. Representative immunoblot images are shown (*n* = 3). (**C**,**E**) Relative protein expression was quantified by densitometry, with Histone H3 as a nuclear loading control (*n* = 3). (**F**) mRNA expression of pro-inflammatory cytokines TNF-α, IL-1β, and IL-6 was determined by qRT-PCR in the indicated groups (*n* = 3). (**G**) Protein expressions of TNF-α, IL-1β, and IL-6 were further validated by Western blot. Representative blots are shown. (**H**,**I**) Semi-quantitative analysis of cytokine protein levels was performed, with GAPDH as the loading control (*n* = 3). Data are presented as mean ± SD. Statistical analysis was performed using one-way ANOVA; * *p* < 0.05, ** *p* < 0.01 versus Mock (OGD/R blank control), siRNA-NC, or pcDNA3.1-NC groups. NF-κB, nuclear factor-kappaB; TNF-α, tumor necrosis factor-α; IL-1β, Interleukin 1 beta.

The NF-κB pathway plays an important role in the overall pathological progression of I/R injury. To investigate the impact of lncRNA-1810026B05Rik on the NF-κB pathway, in vitro models of OGD/R-induced injury were generated. This was achieved by subjecting neurons to a 4 h deprivation of oxygen and glucose, followed by a 12 h recovery period. By transfecting si-1810026B05Rik, a decrease in the levels of lncRNA-1810026B05Rik in OGD/R-treated neurons compared to si-NC. Conversely, transfecting pcDNA3.1-1810026B05Rik increased the expression of lncRNA-1810026B05Rik in OGD/R-treated neurons, in comparison to pcDNA3.1-NC (Figure 4A). Since the activation of NF-κB is localized in the nucleus, the levels of nuclear p65 and p50 were measured by Western blot analysis. The results indicated a substantial increase in the level of nuclear p65 and p50 following OGD/R exposure. Moreover, the overexpression of lncRNA-1810026B05Rik promoted the level of nuclear p65 and p50 in OGD/R-treated neurons, while downregulation of lncRNA-1810026B05Rik yielded the opposite effect (Figure 4B–E). Additionally, the findings exhibited that increased levels of lncRNA-1810026B05Rik intensified the transcription and protein manifestation of genes associated with inflammation, such as TNF-α, IL-1β, and IL-6, in neurons treated with OGD/R. Conversely, the downregulation of lncRNA-1810026B05Rik resulted in decreased levels of genes associated with inflammation (Figure 4F–I). These findings imply that lncRNA-1810026B05Rik promotes the nuclear translocation of p65 and p50, thereby enhancing the level of genes associated with inflammation in neurons.

### 2.5. lncRNA-1810026B05Rik Contributed to IκBα Phosphorylation in OGD/R-Treated Neurons

**Figure 5:**
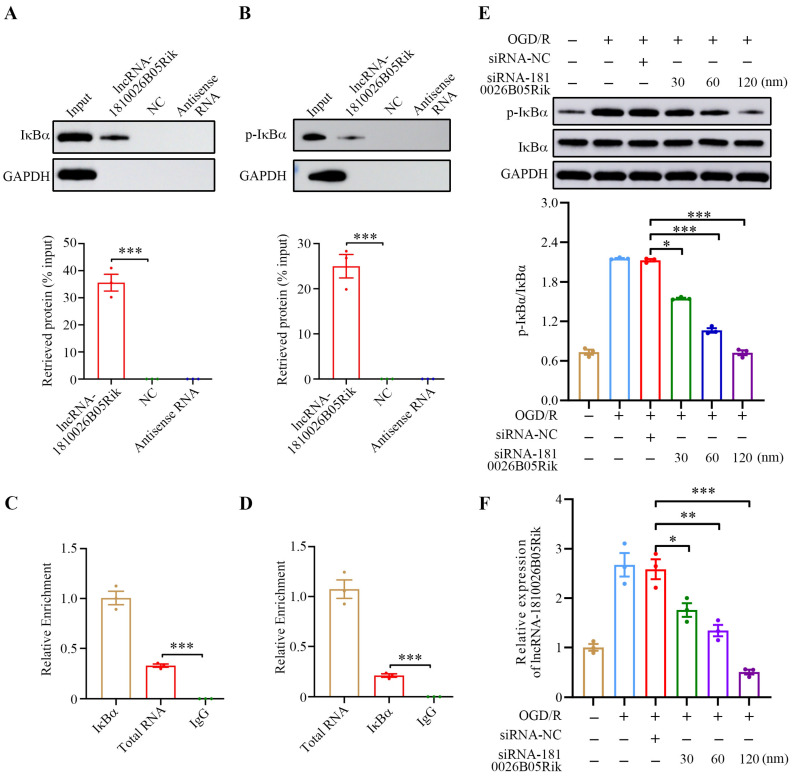
Interaction between lncRNA-1810026B05Rik and NF-κB inhibitor alpha (IκBα). (**A**,**B**) RNA pull-down assays were conducted in OGD/R-treated neurons using biotin-labeled 1810026B05Rik, control RNA, or no RNA. The presence of IκBα and phosphorylated IκBα (p-IκBα) in the pulled-down protein complex was examined via Western blot. Densitometric quantification was normalized to input protein (*n* = 3). (**C**,**D**) RNA immunoprecipitation (RIP) was performed using antibodies against IκBα, p-IκBα, or IgG as a negative control, followed by qRT-PCR to detect enrichment of 1810026B05Rik (*n* = 3). (**E**) Protein levels of IκBα and p-IκBα were detected in the indicated groups by Western blot, and the ratio of p-IκBα/IκBα was quantified (*n* = 3). (**F**) Dose-dependent knockdown efficiency of 1810026B05Rik was validated by qRT-PCR in neurons treated with increasing concentrations (30 nM, 60 nM, and 120 nM) of si-1810026B05Rik, with 50 nM si-NC as control (*n* = 3). All data are represented as mean ± SD. One way ANOVA, * *p* < 0.05, ** *p* < 0.01, *** *p* < 0.001 versus lncRNA-1810026B05Rik, siRNA-NC, Total RNA, IκBα or siRNA-1810026B05Rik group. IκBα, NF-kappa-B inhibitor alpha.

Numerous reports suggest that by interacting with specific proteins, lncRNA exhibits various biological effects. Hence, our hypothesis is that lncRNA-1810026B05Rik potentially modulates the NF-κB signaling pathway by forming a physical association with relevant molecules. Subsequently, this pathway becomes instrumental in carrying out its functional role. To validate this hypothesis, we conducted in vitro RNA pull-down assays on OGD/R-treated neurons using biotinylated lncRNA-1810026B05Rik. Remarkably, it exhibited specific association and substantial precipitation of IκBα protein in comparison to the control groups (Figure 5A). Furthermore, phosphorylated IκBα (Ser32/Ser36) was also found to precipitate significantly (Figure 5B). These findings strongly suggest the physical binding ability of lncRNA-1810026B05Rik towards both IκBα and its phosphorylated counterpart.

To validate the correlation between lncRNA-1810026B05Rik and IκBα, RNA immunoprecipitation (RIP) was utilized in conjunction with anti-IκBα and p-IκBα (Ser32/Ser36) antibodies. Subsequently, the neurons treated with lncRNA-1810026B05Rik in the context of OGD/R were assessed using qRT-PCR. Remarkably, the results illustrated a substantial enrichment of lncRNA-1810026B05Rik within both IκBα and p-IκBα (Figure 5C,D). Consequently, this evidence strongly supports the notion that lncRNA-1810026B05Rik can establish a physical interaction with IκBα under in vivo and in vitro conditions. Moving forward, an inquiry into the biological implications arising from the physical association between lncRNA-1810026B05Rik and IκBα was carried out. In this pursuit, a higher dosage of si-1810026B05Rik was transfected into OGD/R-induced neurons (Figure 5E) and subsequently subjected to evaluate the expression levels of IκBα and p-IκBα (Ser32/Ser36). A significant decrease in p-IκBα/IκBα expression was observed as the lncRNA-1810026B05Rik levels decreased (Figure 5F). The detected results reveal that lncRNA-1810026B05Rik possesses the capability to associate with IκBα and govern the activation of IκBα, subsequently impacting the cascading NF-κB pathway.

### 2.6. Knockdown of lncRNA-1810026B05Rik Functioned as NF-κB Inhibitor and Relieved OGD/R-Induced Cell Death and Necrosis

**Figure 6:**
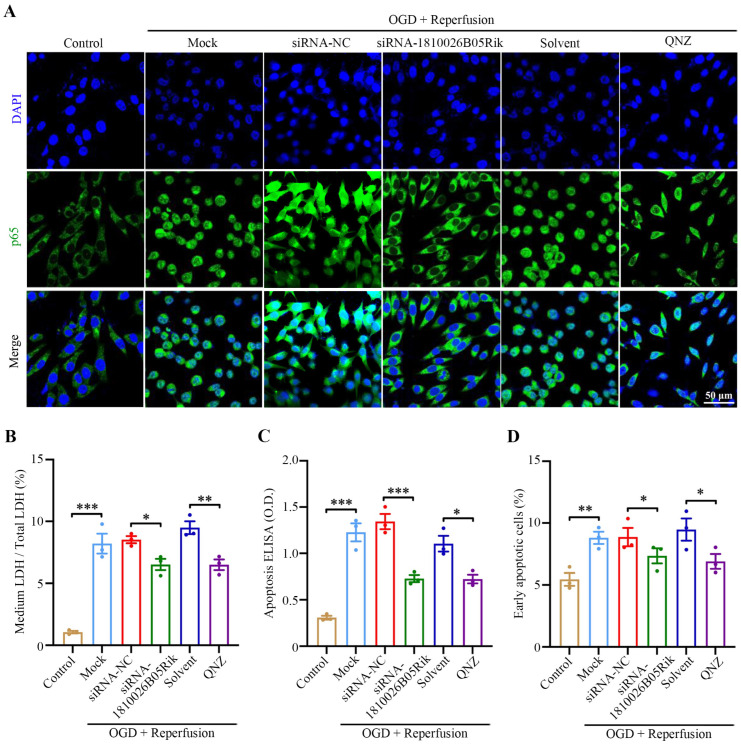
Effects of siRNA-1810026B05Rik and the NF-κB inhibitor QNZ on OGD/R-induced neuronal injury. (**A**) Primary neurons were treated with siRNA-NC, siRNA-1810026B05Rik, QNZ, or ethanol solvent (vehicle control) for 24 h, followed by OGD/R. Immunofluorescence staining was performed to assess p65 localization; nuclei were counterstained with DAPI (blue). Scale bar = 50 μm. (**B**) Neuronal cell death was evaluated using an LDH release assay (*n* = 3). (**C**) Apoptosis was quantified using a Histone DNA ELISA assay (*n* = 3). (**D**) Early apoptotic cells were detected by Annexin V staining and analyzed via flow cytometry (*n* = 3). All values are presented as mean ± SD. Statistical significance was determined by one-way ANOVA; * *p* < 0.05, ** *p* < 0.01, *** *p* < 0.001 vs. Control, siRNA-NC, or Solvent group.

We then proceeded to investigate the impact of lncRNA-1810026B05Rik on neuronal damage caused by OGD/R. The results indicated a substantial increase in the level of nuclear p65 following OGD/R exposure. Moreover, the downregulation of lncRNA-1810026B05Rik decreased the level of nuclear p65 in OGD/R-treated neurons, while treatment with the NF-κB inhibitor QNZ yielded the similar effect (Figure 6A). Our analysis using Western blotting demonstrated that exposure to OGD/R increased the protein levels of cleaved caspase-3/9, and Bax, while inhibiting the level of Bcl-2. However, in neurons, down-regulating lncRNA-1810026B05Rik resulted in decreased expression of cleaved caspase-3/9, and Bax induced by OGD/R and increased levels of Bcl-2. Moreover, when OGD/R neurons were treated with the NF-κB inhibitor QNZ, the LDH release test confirmed that si-1810026B05Rik and QNZ treatment effectively suppressed the death and necrosis of OGD/R neurons (see Figure 6B), the difference observed between the apoptosis ELISA assay and the flow cytometry assay indicated that treatment with lncRNA-1810026B05Rik and QNZ alleviated OGD/R-induced cell apoptosis (Figure 6C,D). Therefore, in comparison with QNZ, these results suggest that downregulation of lncRNA-1810026B05Rik has a NF-κB inhibitory effect against OGD/R-induced damage.

### 2.7. Knockdown of lncRNA-1810026B05Rik Protects Against Cerebral I/R Injury by Activating the NF-κB Pathway

**Figure 7:**
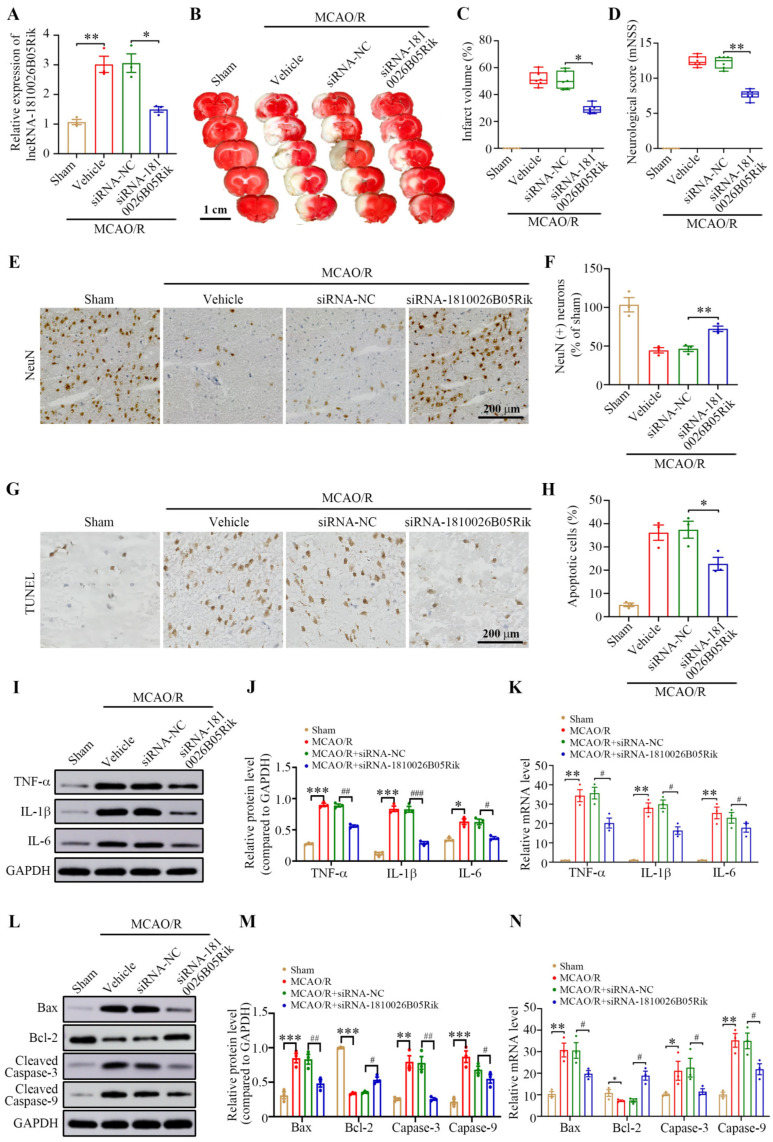
LncRNA-1810026B05Rik aggravated CIRI via activation of the NF-κB pathway. (**A**) Detection of 1810026B05Rik mRNA expression after siRNA-NC and siRNA-1810026B05Rik intracerebroventricular injection of MCAO/R (*n* = 3). (**B**) Representative TTC stained (magenta: healthy tissue; white: damaged tissue) brain tissue sections. Scale bar: 1 cm. (**C**) Quantitative analysis of mouse cerebral infarction volume (*n* = 6). (**D**) Neurological scoring using mNSS after MCAO/R (*n* = 6). lncRNA-1810026B05Rik regulates neuronal survival and inflammation in the ischemic penumbra following MCAO/R. (**E**) Representative immunohistochemistry images of NeuN staining illustrate neuronal loss in the peri-infarct cortex. Scale bar = 200 μm. (**F**) Quantification of neuronal loss in the ischemic penumbra following lncRNA-1810026B05Rik knockdown (*n* = 3). (**G**) TUNEL staining was performed to visualize apoptotic cells in the peri-infarct region. Scale bar = 200 μm. (**H**) Quantitative analysis demonstrated that knockdown of lncRNA-1810026B05Rik significantly elevated neuronal apoptosis in the ischemic cortex (*n* = 3). (**I**–**K**) mRNA and protein levels of pro-inflammatory cytokines TNF-α, IL-1β, and IL-6 were measured 24 h post-reperfusion in mice that underwent 1.5 h of MCAO and received intraventricular injections of either siRNA-NC or siRNA-1810026B05Rik (*n* = 3). (**L**,**M**) Western blotting was used to detect apoptosis-associated proteins Bax, Bcl-2, cleaved caspase-3, and cleaved caspase-9. GAPDH served as a loading control (*n* = 3). (**N**) The transcriptional expression of Bax, Bcl-2, Caspase-3, and Caspase-9 was evaluated via qRT-PCR in neurons transfected with siRNA-NC or siRNA-1810026B05Rik under MCAO/R conditions. Expression levels were normalized to GAPDH (*n* = 3). All values are presented as mean ± SD. Statistical comparisons were performed using one-way ANOVA; * *p* < 0.05, ** *p* < 0.01, *** *p* < 0.001 vs. Sham or Vehicle group; # *p* < 0.05, ## *p* < 0.01, ### *p* < 0.001 vs. MCAO + siRNA-NC group. Bax, BCL2-associated X protein.

To investigate the role of lncRNA-1810026B05Rik in CIRI in vivo, mice were intracortically injected with either siRNA-1810026B05Rik or the negative control siRNA-NC. Forty-eight hours post-injection, the animals were subjected to MCAO/R. Neurological function and cerebral infarct size were assessed 24 h after reperfusion. As depicted in Figure 7A–D, treatment with siRNA-1810026B05Rik observably decreased the infarct volume and alleviated the neurological deficit. We analyzed the histological properties of the cortical penumbra and observed that siRNA-1810026B05Rik treatment markedly reduced neural cell loss, as indicated by anti-NeuN immunohistochemistry data (Figure 7E,F). Furthermore, the TUNEL assay revealed a considerable reduction in MCAO/R-induced neuronal apoptosis following siRNA-1810026B05Rik injection (Figure 7G,H). This suggests that the downregulation of lncRNA-1810026B05Rik can effectively reverse the injury caused by MCAO/R. In order to reveal the underlying mechanism of lncRNA-1810026B05Rik, the expression of downstream inflammatory markers and pro-apoptotic molecules involved in the NF-κB pathway were analyzed using Western blot. The findings revealed a decrease in the levels of genes associated with inflammation upon siRNA-1810026B05Rik treatment after MCAO/R exposure (Figure 7I,J). Figure 7K–N demonstrates that the NF-κB pathway effectively regulates the level of downstream pro-apoptotic factors, which were notably downregulated in mice treated with siRNA-1810026B05Rik following MCAO/R. By inhibiting lncRNA-1810026B05Rik, the level of the antiapoptotic factor Bcl-2 was increased. These results suggest that knocking down lncRNA-1810026B05Rik may provide protection against MCAO/R-induced injury by inhibiting activation of the NF-κB pathway.

## 3. Discussion

Numerous studies have established that inflammation and apoptosis induction are significantly driven by the NF-κB signal transduction pathway. The activation of NF-κB transcription plays a critical role in regulating various pro-inflammatory cytokines, as well as apoptosis-related factors [28]. The central nervous system relies on neurons to function normally and facilitate the transmission of signal instructions, making them vital in instances of central nervous system injury [29]. Furthermore, past investigations have presented definitive proof demonstrating that the NF-κB pathway activation is excessive in neurons undergoing CIRI [30,31]. In this investigation, we show that OGD/R (oxygen-glucose deprivation/reoxygenation) and MCAO/R (middle cerebral artery occlusion/reperfusion) treatments trigger the activation of the NF-κB pathway in neurons, leading to increased expression of pro-inflammatory cytokines and apoptotic factors like Bax and Caspase-3. This aligns with existing literature indicating that the activation of NF-κB in neurons during ischemia/reperfusion plays a pivotal role in driving the inflammatory and apoptotic responses that contribute to tissue damage. Furthermore, our results suggest that the NF-κB pathway is a major contributor to the progression of ischemic injury in neurons, which underscores the therapeutic potential of targeting NF-κB signaling in stroke treatment. While we have confirmed the activation of NF-κB, further research is needed to identify specific mechanisms that lead to this activation and to determine how it can be controlled in therapeutic settings.

Recent studies have demonstrated that lncRNA participates in various signal transduction and regulates downstream gene transcription through trans or cis effects [12]. Therefore, this study explored whether lncRNA is involved in cerebral ischemia/reperfusion-mediated NF-κB activation. Chaserr, an undocumented lncRNA referred to as lncRNA-1810026B05Rik in mice, and LINC01578/LOC100507217 in humans (known as CHASERR), exerts influence on multiple cellular phenomena such as cell differentiation, apoptosis, development, and tumorigenesis. This lncRNA is situated upstream of CHD2, transcribed from the same strand, and exhibits suppressive regulatory characteristics [32]. We found that lncRNA-1810026B05Rik is significantly upregulated after ischemic injury in both in vivo and in vitro models. The temporal increase in lncRNA-1810026B05Rik expression at 12 h post-MCAO/R or OGD/R suggests that it plays a critical early role in mediating cellular responses to ischemia. This finding aligns with other studies that have shown increased lncRNA expression during ischemic events in both animal models and human stroke patients [16]. The present investigation uncovered that the regulation of lncRNA-1810026B05Rik implies its potential involvement in the I/R injury. Our findings exhibited that the expression of lncRNA-1810026B05Rik started augmenting at the onset of 12 h following MCAO/R or OGD/R, and eventually diminished within the time span of 24–48 h. Furthermore, the overexpression of lncRNA-1810026B05Rik in OGD/R-treated neurons induced an upsurge in the level of nuclear P65/P50 and triggered the excessive transcriptional activation of TNF-α, IL-6 and IL-1β. This further corroborates the hypothesis that lncRNAs can regulate inflammation and apoptosis during ischemia/reperfusion. Moreover, the interaction of lncRNA-1810026B05Rik with IκBα—an essential regulator of NF-κB activation—suggests that lncRNA-1810026B05Rik may directly influence the phosphorylation and degradation of IκBα, thereby facilitating the nuclear translocation of NF-κB subunits and enhancing the inflammatory response. These findings are consistent with prior reports, where lncRNAs have been shown to interact with key signaling proteins like IκBα and influence NF-κB activity during inflammatory responses. Interestingly, our study highlights that the knockdown of lncRNA-1810026B05Rik alleviates the inflammatory response and mitigates neuronal apoptosis. Specifically, in cellular models, knocking down lncRNA-1810026B05Rik reduced the nuclear levels of p65 and p50, which correlated with decreased expression of pro-inflammatory cytokines and apoptotic factors. This neuroprotective effect observed upon silencing lncRNA-1810026B05Rik further supports the potential of targeting this lncRNA to attenuate ischemic damage. Moreover, our data suggest that inhibiting lncRNA-1810026B05Rik could be a viable strategy to reduce ischemic injury, particularly through NF-κB modulation. The concept of using lncRNA as therapeutic targets in ischemic stroke is gaining traction, as shown in studies exploring lncRNA interactions in other inflammatory diseases.

Scientific research has previously demonstrated that disease inflammation, fibrosis, and cell proliferation are greatly facilitated by the NF-κB signaling pathways. Comprising five members, namely NF-κB1 (P50), NF-κB2 (P52), RelA (P65), RelB, and C-REL, the NF-κB family plays a significant role in these processes [33]. Upon exposure to various stressors, the NF-κB P65/P50 that is up-regulated binds to the promoter regions of mRNA or lncRNA. As a consequence, it triggers transcriptional activation leading to the initiation of a positive feedback loop [34]. Our founding has confirmed that lncRNA-1810026B05Rik activates the NF-κB pathway and mediates downstream inflammatory apoptotic responses induced by cerebral I/R. However, the mechanism of lncRNA-1810026B05Rik in the activation of NF-κB remains elusive. Our study demonstrated that lncRNA-1810026B05Rik can bind to IκBα, and the reduced level of lncRNA-1810026B05Rik downregulates phosphorylated IκBα (Ser32/Ser36). The provided data suggests that lncRNA-1810026B05Rik collaborates with IκBα to enhance the phosphorylation of IκBα at Ser32 and Ser36. It is widely acknowledged that NF-κB activation occurs through the phosphorylation of IκBα at Ser32 and Ser36, leading to subsequent nuclear translocation and transcriptional activation of target genes [35,36]. Further investigations using co-immunoprecipitation, mass spectrometry, and advanced proteomic techniques could provide deeper insights into the protein interactome involved with lncRNA-1810026B05Rik and its precise role in NF-κB activation. Additionally, while we focused on lncRNA-1810026B05Rik’s interaction with IκBα, it is crucial to explore the involvement of other lncRNAs in modulating NF-κB during ischemic injury, particularly as NF-κB is a key player in many inflammatory diseases. Furthermore, the down-regulation of lncRNA-1810026B05Rik has the potential to function as an NF-κB inhibitor, leading to the enhancement of OGD/R injury recovery and the mitigation of neuronal apoptosis and demise. As a result, lncRNA-1810026B05Rik, which serves as a pivotal modulator of NF-κB signaling, displays promising efficacy as a therapeutic target for combating OGD/R injury. We will further confirm the role of lncRNA-1810026B05Rik by constructing a genetic model of lncRNA-1810026B05Rik knockout mice, which also promotes the understanding of lncRNA-1810026B05Rik as a potential target during clinical transformation.

Our study suggests that the downregulation of lncRNA-1810026B05Rik acts as an effective NF-κB inhibitor, promoting recovery from ischemic injury and reducing neuronal apoptosis. Thus, lncRNA-1810026B05Rik represents a promising therapeutic target for mitigating cerebral ischemia/reperfusion injury. To further validate its therapeutic potential, we plan to construct a genetic model of lncRNA-1810026B05Rik knockout mice. This will provide more insights into the role of lncRNA-1810026B05Rik in vivo and strengthen the foundation for its potential clinical application.

## 4. Materials and Methods

### 4.1. Animal and Focal Cerebral Ischemia

All animal procedures were approved by the Animal Ethics Committee of Guangzhou Huateng Biomedical Technology Co., Ltd., Guangzhou, China (Approval No. HTSW221143, approved on 19 April 2023). Male C57BL/6 mice (7–8 weeks old, weighing 21–23 g) were obtained from the Animal Research Centre of Guangzhou University of Chinese Medicine. Prior to experimentation, mice were acclimated under standardized conditions: a temperature-controlled environment (20 ± 2 °C) with a 12 h light/dark cycle, and free access to food and water. Transient focal cerebral ischemia was induced using the MCAO model based on the protocol described by Li et al. [37]. Mice were subjected to 90 min of ischemia followed by reperfusion for 0, 6, 12, 24, or 48 h. Sham-operated animals underwent the same surgical steps without filament insertion to induce occlusion.

### 4.2. In Vivo lncRNA-1810026B05Rik Down Expression

The siRNA-1810026B05Rik that were acquired from RiboBio were utilized, with siRNA-NC serving as the negative control (NC) (Guangzhou, China). To anesthetize the mice, a stereotactic apparatus was employed, and 1.5–3.0% isoflurane was administered. The coordinates employed for the stereotaxic procedure were as follows: anteroposterior = 0.8 mm, mediolateral = ±1.4 mm, and dorsoventral = 3.5 mm with respect to bregma. Administering the plasmids containing siRNA-1810026B05Rik or shRNA-NC was achieved via slow injection over a span exceeding 15 min into the lateral ventricles. Two days were allotted to enable the mice to recover and facilitate subsequent lncRNA-1810026B05Rik knockdown. After the transfection had occurred for two days, the mice underwent MCAO for further studies.

### 4.3. Neurological Function and Infarct Volume Evaluation

Neurobehavioral impairments following reperfusion were evaluated on the first day by employing the revised Neurological Severity Score (mNSS). Mice were sacrificed 24 h later, and brain sections were generated through application of the 1.0% triphenyl tetrazolium chloride (TTC) staining to quantify the volume of infarction [38,39].

### 4.4. Cell Culture and OGD Treatment

Cultured neurons derived from the brain cortex of postnatal day 1–2 C57BL/6 mice were isolated to study the impact of in vitro I/R injury. To mimic this injury, an OGD treatment was administered to the neurons. Subsequently, the cells were returned to their regular conditions and allowed to recover for 0, 6, 12, 24, and 48 h. As a comparison, a group of cultured neurons was kept under identical conditions but without exposure to the OGD.

### 4.5. Cell Transfection

siRNAs targeting lncRNA-1810026B05Rik (siRNA-1810026B05Rik) and a corresponding negative control (siRNA-NC), as well as the overexpression vector pcDNA3.1-1810026B05Rik (pcDNA3.1-1810026B05Rik) and its control pcDNA3.1vector (pcDNA3.1-NC), were obtained by RiboBio Co., Ltd. (Guangzhou, China). Following appropriate cell seeding, cultured neurons were transfected following the protocols provided by the manufacturer. Subsequent experiments were performed 24 h after transfection, and neurons were collected for analysis [2].

### 4.6. RNA Fluorescence In Situ Hybridization (RNA FISH)

To carry out the FISH assay, we followed the guidelines provided by the manufacturer to create fluorescence-conjugated probes specific to lncRNA-1810026B05Rik. Neurons were cultured, followed by the hybridization step with the lncRNA-1810026B05Rik probes (RiboBio, Guangzhou, China). Subsequently, the neurons were incubated with DAPI for a duration of 10 min. The resulting samples were then observed using confocal microscopy (Axio Image A2, Zeiss, Oberkochen, Germany).

### 4.7. Cell Viability Assay

To assess cell viability, 10 μL of CCK-8 reagent (Beyotime, Haimen, Jiangsu, China) was added to each well, followed by a 2 h incubation at 37 °C. Subsequently, 150 μL of dimethyl sulfoxide (DMSO) was added to dissolve the formazan product. The absorbance was measured at 450 nm using a microplate reader (WoYuan, Shanghai, China). Cell viability was expressed as a percentage relative to the untreated control group (no OGD/R exposure).

### 4.8. Cell Apoptosis and Death Assays

DNA fragment detection was performed by the ELISA method (apoptosis-ELISA method) to detect cell apoptosis [40]. A specific two-site ELISA method was used in combination with anti-histone primary antibodies and anti-DNA secondary antibodies. The determination of nucleosome histone-related DNA was carried out following the instructions (Roche). Neurons were gathered and subjected to double staining using fluorescein isothiocyanate (FITC)-coupled Annexin V and iodinated precursor (PI), employing an apoptosis detection kit. Flow cytometry was utilized to assess the percentage of neurons undergoing early apoptosis. This experiment was conducted thrice. For the cell death assessment, the LDH kit (Thermo Fisher Scientific, Waltham, MA, USA) was employed to quantify the amount of LDH released from the cells into the culture medium, indicating the level of toxicity, as previously described in the literature [41].

### 4.9. Measurement of Intracellular Reactive Oxygen Species (ROS) Levels

The ROS levels can be achieved by analyzing the nitrate content present in the culture supernatants [42]. To fulfill this objective, dichloro-dihydro-fluorescein diacetate (DCFH-DA) (Thermo Fisher Scientific, Waltham, MA, USA) was diluted with medium, resulting in a final concentration of 5 μM. Following an incubation period of 60 min in the dark, the culture supernatant derived from neuronal cells was collected and subjected to examination by confocal microscope (Axio Image A2, Zeiss, Oberkochen, Germany). The absorbance rate at 550 nm was subsequently measured to quantify the findings.

### 4.10. Determination of Intracellular Ca^2+^ of Neurons

Intracellular Ca^2+^ levels were measured using the fluorescent calcium indicator Fluo-4 AM (Beyotime, Shanghai, China) in accordance with the manufacturer’s instructions. Cells were washed twice with PBS and incubated at 37 °C in the dark for 30 min in a solution containing 10 mmol/L Fluo-4 AM and 5 mg/mL Hoechst 33342. Fluorescence images were captured using a Zeiss Axio Imager A2 fluorescence microscope, and calcium signal intensity was quantified using ImageJ software 1.50 (NIH, Bethesda, MD, USA) to assess relative intracellular Ca^2+^ concentrations.

### 4.11. TUNEL Staining

We employed a TUNEL assay to identify and quantify neuronal apoptosis. To begin, cells or brain tissue fractions were affixed to a slide using ice-cold 4% paraformaldehyde for a duration of 10 min. Subsequently, permeabilization was achieved using 0.1% (*v*/*v*) Triton X-100 (Sigma, St. Louis, MO, USA) for 5 min. We visualized and captured images of positive TUNEL staining (Sigma, St. Louis, MO, USA) using a fluorescence microscope. Lastly, we determined the apoptotic index, which represents the percentage of TUNEL-positive cells relative to the total cell count (% positive cells/100% total cells).

### 4.12. RNA-Binding Protein Immunoprecipitation (RIP) Assay

The performance of RNA pull-down and RIP was carried out following the methods described in previous studies [43,44]. Biotin-labeled RNA was transcribed by biotin RNA-labeled hybrid (Thermo Fisher Scientific, Waltham, MA, USA) and T7 RNA polymerase in vitro. A blend of cell extract and biotinylated RNA was combined, and subsequently, the mixture was exposed to washed streptavidin agarose beads. Following this, an analysis using Western blot was conducted in order to ascertain the protein that had been extracted. In order to carry out the RIP detection, a kit specifically designed was utilized (Millipore, Billerica, MA, USA), along with p-IκBa and IκBa antibodies from Abcam, or a negative IgG control (ab133470; Abcam). Subsequently, the RNA that had been bound was separated from the levels of lncRNA-1810026B05Rik were measured using qRT-PCR.

### 4.13. Quantitative Real-Time Polymerase Chain Reaction (qRT-PCR)

We utilized the manufacturer’s protocols to extract Total RNA, employing TRIzol reagent. Transcription was conducted for a duration of 30 min, followed by incubation (Vazyme, Nanjing, China). The enzyme was then inactivated at a temperature of 85 °C for 5 min. These reactions were executed utilizing the subsequent parameters: 16 °C for a duration of 30 min, 42 °C for a timeframe of 30 min, and 84 °C for 5 min. The reaction of qRT-PCR was completed employing the ensuing parameters: 95 °C for 2 min, followed by 40 cycles of 95 °C for 10 s and 60 °C for 20 s. We standardized all the outcomes to the expression of GAPDH. We performed quantitative analysis using the 2^−ΔΔCt^ method. The utilized primer sequences were as follows: TNF-α, 5′-CCTCACACTCAGATCATCTTCT-3′ and 5′-GCTACGACGTGGGCTACAG-3′; IL-1β, 5′-GCAACTGTTCCTGAACTCAACT-3′ and 5′-ATCTTTTGGGGTCCGTCAACT-3′; IL-6, 5′-TAGTCCTTCCTACCCCAATTTCC-3′ and 5′-TTGGTCCTTAGCCACTCCTTC-3′; Bax, 5′-GCTGATGGCAACTTCAACTG-3′ and 5′-GATCAGCTCGGGCACTTTAG-3′; Bcl-2, 5′-CCAGCGTGTGTGTGCAAGTGTAAAT-3′ and 5′-ATGTCAATCCGTAGGAATCCCAACC-3′; caspase-3, 5′-AGATACCGGTGGAGGCTGACT-3′ and 5′-TCTTTCGTGAGCATGGACACA-3′; caspase-9, 5′-ATCAACAGGCTATCCGGAACC-3′ and 5′-CTTATCACCGTCCCTTCT-3′; 1810026B05Rik, 5′-TGTGGCTCCCAAGTGAAG-3′ and 5′-GAGCACCAGTACACCAAAG-3′; and GAPDH, 5′-AACGATTTGGTTATTG-3′ and 5′-GGAAGATGTGGTATT-3′.

### 4.14. Western Blotting

Total proteins were extracted from neuronal cells and brain tissues using conventional protocols. Nuclear fractions were isolated using a nuclear/cytoplasmic protein extraction kit. Equal amounts of protein were separated by SDS-PAGE and transferred onto PVDF membranes. Membranes were incubated overnight at 4 °C with primary antibodies (1:1000), including rabbit monoclonal antibodies against p-IκBα (ab133462), IκBα (ab32518), Bcl-2 (ab32124), cleaved Caspase-3 (CST9579), cleaved Caspase-9 (CST9509), TNF-α (ab205587), IL-1β (ab283818), IL-6 (ab259341), rabbit polyclonal antibodies against Bax (ab182733), and GAPDH (ab9485). All antibodies were purchased from Abcam unless otherwise specified. After washing, membranes were incubated with HRP-conjugated goat anti-rabbit IgG secondary antibody (ab205718, 1:2000). Protein bands were visualized using X-ray film, and densitometric analysis was performed using ImageJ software (NIH, Bethesda, MD, USA).

### 4.15. Statistical Analysis

To analyze the data statistically, we employed SPSS 18.0 software (IBM Corp, Armonk, NY, USA), which allowed us to conduct either a Student’s *t*-test or one-way ANOVA. Through this analysis, we were able to gain insights into the findings gathered from three separate experiments. The data collected from these experiments were then presented as the mean (x) ± standard deviation (SD), providing a more accurate representation of the overall results. A *p*-value < 0.05 was considered statistically significant.

## 5. Conclusions

**Figure 8:**
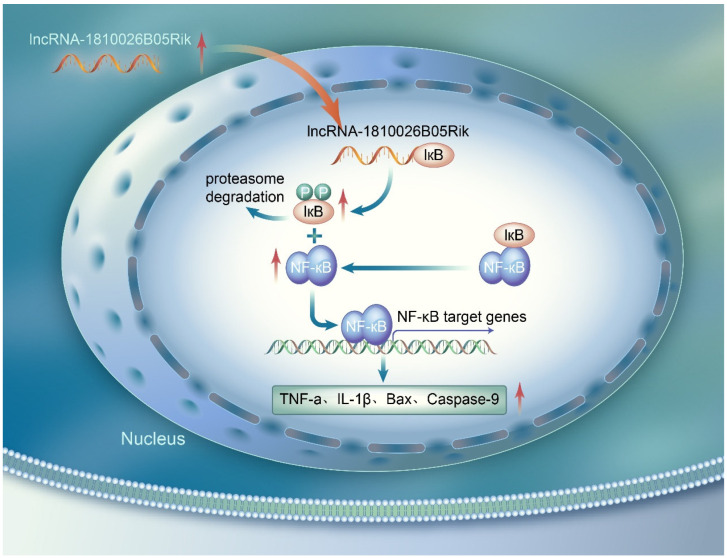
Experimental mechanism diagram 1810026B05Rik, originally exists in the cytoplasm, transports into the nucleus with elevated expression upon cerebral ischemia/reperfusion injury. 1810026B05Rik combined with IκBα and resulted in its phosphorylation. The phosphorylated IκBα promoted the neural expression of p65 and p50 in nucleus and activated NF-κB signaling pathway, which further enhanced downstream expression of TNF-α, IL-1β, Bax and casapse-9. The secretion of pro-inflammatory and proapoptotic factors aggravated neural inflammatory reaction and apoptosis, exacerbating the progression of disease.

In this study, we propose a novel role for the lncRNA-1810026B05Rik/IκBα axis in neurons following cerebral ischemia/reperfusion injury (CIRI). These results reveal that lncRNA-1810026B05Rik promotes the transcription of downstream inflammatory and apoptotic genes by phosphorylating IκBα and activating the NF-κB pathway, which exacerbates the neuronal necrosis induced by CIRI (Figure 8). Our findings suggest that lncRNA-1810026B05Rik is a critical modulator of neuroinflammation and neuronal apoptosis, making it a promising therapeutic target for ischemic stroke. The results indicate that modulating the expression of lncRNA-1810026B05Rik could provide significant neuroprotective effects. However, further research, including clinical trials and additional animal models, is required to fully understand its therapeutic potential. Despite the promising results, there are several limitations to this study. First, the study was conducted primarily in animal models, and the findings need to be validated in human studies to confirm their translational potential. Additionally, we did not incorporate certain assays, such as the comet assay, which could have provided more definitive evidence of DNA fragmentation. Time and resource constraints prevented the inclusion of these methods, but we plan to address these limitations in future studies. Finally, while we focused on the role of lncRNA-1810026B05Rik, the involvement of other lncRNAs and their potential interactions with this pathway were not explored, which warrants further investigation.

## Data Availability

The original contributions presented in this study are included within this article Materials. Further inquiries can be directed to the corresponding authors.

## References

[1] Behrouzifar S., Vakili A., Bandegi A.R., Kokhaei P. (2018). Neuroprotective nature of adipokine resistin in the early stages of focal cerebral ischemia in a stroke mouse model. Neurochem. Int..

[2] Zhang H., Lu M., Zhang X., Kuai Y., Mei Y., Tan Q., Zhong K., Sun X., Tan W. (2019). Isosteviol sodium protects against ischemic stroke by modulating microglia/macrophage polarization via disruption of GAS5/miR-146a-5p sponge. Sci. Rep..

[3] Singh N., Santos T., Ali A.B., Khan H., Kibrik P., Storch J., Bai H., Awad M., Patel R., Huber M. (2025). Contraindications to tissue plasminogen activator thrombolysis for acute lower extremity ischemia. Vascular.

[4] Khanra S., Paul N., Mukherjee S. (2018). Early Marked Behavioral Symptoms in Bilateral Posterior Cerebral Artery Stroke: A Disguised Presentation. Indian J. Psychol. Med..

[5] Pan Q., Liu Y., Wang G., Wen Z., Wang Y. (2020). MTMR14 protects against cerebral stroke through suppressing PTEN-regulated autophagy. Biochem. Biophys. Res. Commun..

[6] Akıl E., Akıl M.A., Varol S., Özdemir H.H., Yücel Y., Arslan D., Akyüz A., Alan S. (2014). Echocardiographic Epicardial Fat Thickness and Neutrophil to Lymphocyte Ratio Are Novel Inflammatory Predictors of Cerebral Ischemic Stroke. J. Stroke Cerebrovasc. Dis. Off. J. Natl. Stroke Assoc..

[7] Fesenko I.A., Kirov I.V., Filippova A.A. (2018). Impact of Noncoding Part of the Genome on the Proteome Plasticity of the Eukaryotic Cell. Russ. J. Bioorganic Chem..

[8] Takemata N., Ohta K. (2017). Role of non-coding RNA transcription around gene regulatory elements in transcription factor recruitment. RNA Biol..

[9] Mercer T.R., Mattick J.S. (2013). Structure and function of long noncoding RNAs in epigenetic regulation. Nat. Struct. Mol. Biol..

[10] Tao H., Yang J.J., Shi K.H. (2015). Non-coding RNAs as direct and indirect modulators of epigenetic mechanism regulation of cardiac fibrosis. Expert. Opin. Ther. Targets.

[11] Hauptman N., Glava? D. (2013). Long Non-Coding RNA in Cancer. Int. J. Mol. Ences.

[12] Zhang H., Liu B., Shi X., Sun X. (2020). Long noncoding RNAs: Potential therapeutic targets in cardiocerebrovascular diseases. Pharmacol. Ther..

[13] Mirzajani S., Ghafouri-Fard S., Habibabadi J.M., Arsang-Jang S., Omrani M.D., Fesharaki S.S.H., Sayad A., Taheri M. (2020). Expression Analysis of lncRNAs in Refractory and Non-Refractory Epileptic Patients. J. Mol. Neurosci..

[14] Zhu W., Tian L., Yue X., Liu J., Fu Y., Yan Y. (2019). LncRNA Expression Profiling of Ischemic Stroke During the Transition From the Acute to Subacute Stage. Front. Neurol..

[15] Barangi S., Hayes A.W., Reiter R., Karimi G. (2019). The therapeutic role of long non-coding RNAs in human diseases: A focus on the recent insights into autophagy. Pharmacol. Res..

[16] Zhang J., Yuan L., Zhang X., Hamblin M.H., Zhu T., Meng F., Li Y., Chen Y.E., Yin K.J. (2016). Altered long non-coding RNA transcriptomic profiles in brain microvascular endothelium after cerebral ischemia. Exp. Neurol..

[17] Sances S., Ho R., Vatine G., West D., Laperle A., Meyer A., Godoy M., Kay P.S., Mandefro B., Hatata S. (2018). Human iPSC-Derived Endothelial Cells and Microengineered Organ-Chip Enhance Neuronal Development. Stem Cell Rep..

[18] Turner M., Galloway A., Vigorito E. (2014). Noncoding RNA and its associated proteins as regulatory elements of the immune system. Nat. Immunol..

[19] Meller V.H., Joshi S.S., Deshpande N. (2015). Modulation of Chromatin by Noncoding RNA. Annu. Rev. Genet..

[20] Rutenbergschoenberg M., Sexton A.N., Simon M. (2016). The Properties of Long Noncoding RNAs That Regulate Chromatin. Annu. Rev. Genom. Hum. Genet..

[21] Yamamoto E., Tamamaki N., Nakamura T., Kataoka K., Tokutomi Y., Dong Y.F., Fukuda M., Matsuba S., Ogawa H., Kim-Mitsuyama S. (2008). Excess salt causes cerebral neuronal apoptosis and inflammation in stroke-prone hypertensive rats through angiotensin II-induced NADPH oxidase activation. Stroke J. Cereb. Circ..

[22] Tian G.H., Tao S.S., Chen M.T., Li Y.S., Li Y.P., Shang H.C., Tang X.Y., Chen J.X., Tang H.B. (2016). Electroacupuncture Treatment Alleviates Central Poststroke Pain by Inhibiting Brain Neuronal Apoptosis and Aberrant Astrocyte Activation. Neural Plast..

[23] Zhe X., Zhang S., Ren H. (2024). Effect of intravenous thrombolysis with butylphthalide, edaravone and recombinant tissue plasminogen activator (rt-PA) on serum inflammatory factors in patients with ischemic stroke. Trop. J. Pharm. Res..

[24] Meng Q.T., Chen R., Chen C., Su K., Li W., Tang L.H., Liu H.M., Xue R., Sun Q., Leng Y. (2017). Transcription factors Nrf2 and NF-κB contribute to inflammation and apoptosis induced by intestinal ischemia-reperfusion in mice. Int. J. Mol. Med..

[25] Su L., Zhang R., Chen Y., Zhu Z., Ma C. (2017). Raf Kinase Inhibitor Protein Attenuates Ischemic-Induced Microglia Cell Apoptosis and Activation Through NF-κB Pathway. Cell. Physiol. Biochem. Int. J. Exp. Cell. Physiol. Biochem. Pharmacol..

[26] Venna V.R., Weston G., Benashski S.E., Tarabishy S., Liu F., Li J., Conti L.H., Mccullough L.D. (2012). NF-κB contributes to the detrimental effects of social isolation after experimental stroke. Acta Neuropathol..

[27] Cui L., Duchamp N.S., Boston D.J., Ren X., Zhang X., Hu H., Zhao L.R. (2015). NF-κB is involved in brain repair by stem cell factor and granulocyte-colony stimulating factor in chronic stroke. Exp. Neurol..

[28] Yan J., Li T., Ji K., Zhou X., Yao W., Zhou L., Huang P., Zhong K. (2024). Safranal alleviates pentetrazole-induced epileptic seizures in mice by inhibiting the NF-κB signaling pathway and mitochondrial-dependent apoptosis through GSK-3β inactivation. J. Ethnopharmacol..

[29] Wu Z., Song Y., Wang Y., Zhou H., Chen L., Zhan Y., Li T., Xie G., Wu H. (2024). Biological role of mitochondrial TLR4-mediated NF-κB signaling pathway in central nervous system injury. Cell Biochem. Funct..

[30] Liu H., Wu X., Luo J., Wang X., Guo H., Feng D., Zhao L., Bai H., Song M., Liu X. (2019). Pterostilbene Attenuates Astrocytic Inflammation and Neuronal Oxidative Injury After Ischemia-Reperfusion by Inhibiting NF-κB Phosphorylation. Front. Immunol..

[31] Liang W., Lin C., Yuan L., Chen L., Guo P., Li P., Wang W., Zhang X. (2019). Preactivation of Notch1 in remote ischemic preconditioning reduces cerebral ischemia-reperfusion injury through crosstalk with the NF-κB pathway. J. Neuroinflamm..

[32] Rom A., Melamed L., Gil N., Goldrich M.J., Kadir R., Golan M., Biton I., Perry R.B., Ulitsky I. (2019). Regulation of CHD2 expression by the Chaserr long noncoding RNA gene is essential for viability. Nat. Commun..

[33] Anilkumar S., Wright-Jin E. (2024). NF-κB as an inducible regulator of inflammation in the central nervous system. Cells.

[34] Yi H., Peng R., Zhang L.-y., Sun Y., Peng H.-m., Liu H.-d., Yu L.-j., Li A.-l., Zhang Y.-j., Jiang W.-h. (2017). LincRNA-Gm4419 knockdown ameliorates NF-κB/NLRP3 inflammasome-mediated inflammation in diabetic nephropathy. Cell Death Dis..

[35] Foulds C.E., Panigrahi A.K., Coarfa C., Lanz R.B., O’Malley B.W. (2016). Long Noncoding RNAs as Targets and Regulators of Nuclear Receptors. Curr. Top. Microbiol. Immunol..

[36] Howell J.A., Bidwell G.L. (2020). Targeting the NF-κB pathway for therapy of ischemic stroke. Ther. Deliv..

[37] Li X., Ding H., Jing J., Qian S., Ma Y., Lv M., Gao Y., Zhang Y., Li T. (2025). Sulfasalazine improves neuronal function in mice with ischemic stroke by inhibiting the STING/NF-κB pathway. Naunyn-Schmiedeberg’s Arch. Pharmacol..

[38] He L., Lei R., Li S., Zhao X., He X., Yang X., Liu P., Zhang D., Jiang Y. (2025). Hirudin promotes cerebral angiogenesis and exerts neuroprotective effects in MCAO/R rats by activating the Wnt/β-catenin pathway. J. Stroke Cerebrovasc. Dis..

[39] Zhang Q., Han S., Zheng Y., Li J., Ma L., Tan J., Kang X., Gong R., Chen S., Shi S. (2025). The mechanism of promoting angiogenesis after cerebral infarction by scalp acupuncture based on MATN2/WNT3a/β-catenin. J. Mol. Histol..

[40] Gu D.M., Lu P.H., Zhang K., Xiang W., Wang Q. (2015). EGFR mediates astragaloside IV-induced Nrf2 activation to protect cortical neurons against in vitro ischemia/reperfusion damages. Biochem. Biophys. Res. Commun..

[41] Wen Y., Yu Y., Fu X. (2017). LncRNA Gm4419 contributes to OGD/R injury of cerebral microglial cells via IκB phosphorylation and NF-κB activation. Biochem. Biophys. Res. Commun..

[42] Guo Y., Liu J., Du X., Qi M., She T., Xue K., Wu X., Xu L., Peng B., Zhang Y. (2024). ROS exhaustion reverses the effects of hyperbaric oxygen on hemorrhagic transformation through reactivating microglia in post-stroke hyperglycemic mice. Sci. Rep..

[43] Luo M., Li Z., Wang W., Zeng Y., Liu Z., Qiu J. (2013). Long non-coding RNA H19 increases bladder cancer metastasis by associating with EZH2 and inhibiting E-cadherin expression. Cancer Lett..

[44] Luo J., Wang K., Yeh S., Sun Y., Liang L., Xiao Y., Xu W., Niu Y., Cheng L., Maity S.N. (2019). LncRNA-p21 alters the antiandrogen enzalutamide-induced prostate cancer neuroendocrine differentiation via modulating the EZH2/STAT3 signaling. Nat. Commun..

